# Gigant Transethmoidal Meningoencephalocele Operated by Full Endonasal Endoscopic Approach: Case Report

**DOI:** 10.1155/2012/763259

**Published:** 2012-02-21

**Authors:** Omar Lopez Arbolay, Jorge Rojas Manresa, Justo Gonzalez Gonzalez, Jose Luis Bretón Rosario

**Affiliations:** Neurosurgery Department of Hermanos Ameijeiras Hospital, Havana 10348, Cuba

## Abstract

Intranasal meningoencephaloceles have historically been managed by neurosurgeons, although their main clinical manifestations are rhinological. Recent advances in endoscopic skull base surgery has significantly improved the treatment of these lesions and consequently diminished appreciable surgical morbidity. We report an ethmoidal meningoencephalocele case operated on by endonasal endoscopic approach for removal of the lesion and reconstructing the associated skull base. From this experience, we conclude that removal of the lesion and watertight closure of the skull base irrespective of the size of the mass and anterior skull base defect are the operation's most important aspects.

## 1. Introduction

Intranasal meningoencephaloceles are an infrequent condition characterized by protrusion of meningeal and brain tissue through a skull-base defect. Congenital anomalies are the main cause [1], but they may have also a traumatic or a spontaneous origin [2, 3]. According to the location, meningoencephaloceles are classified in: occipital, cranial vault, posterior fosse, and basal. The incidence of this rare condition range between 0.1 to 0.5 of 1,000 birthrates. Basal meningoencephalocele represents 1.5% of all these lesions, and are classified as: transethmoidal, sphenoethmoidal, transsphenoidal, and frontosphenoidal [4, 5]. In transethmoidal type, a defect on the cribriform plate is observed, commonly small and limited to one side [6]. Nasal CSF leakage, headache, and nasal obstruction, are frequently the chief complaints. Rarely, seizure is observed but meningitis is common.

On physical examination, it is possible to find an intranasal mass, leakage of a clear liquid across the nose, olfaction lost, and other craniofacial defects in the congenital encephaloceles. Some studies may be performed to establish the diagnosis, but magnetic resonance imaging (MRI) is the leading investigation because it is able to show the protruded brain tissues and their relationship with neighboring structures. A special useful test to demonstrate the bony defect will be the CT scan with bone window [1]. 

We present a case with transethmoidal meningoencephalocele that was operated on by endonasal endoscopic technique.

## 2. Clinical Case

**Figure 1:**
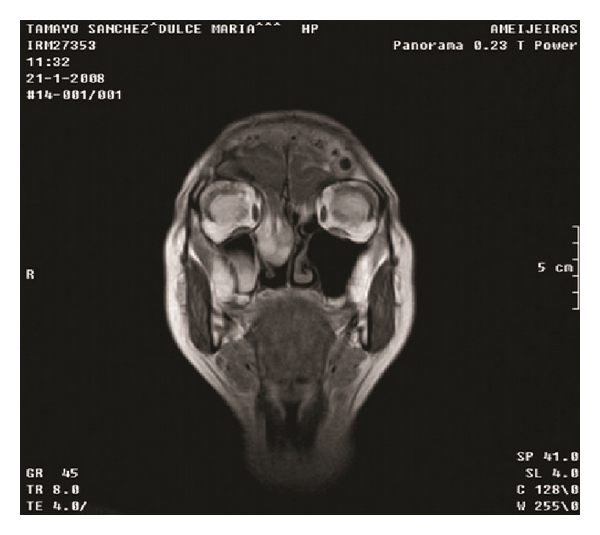
MRI, coronal view.

**Figure 2:**
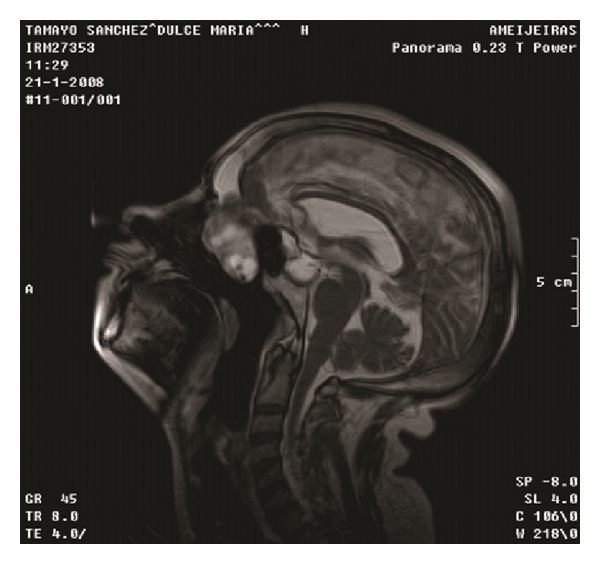
MRI, sagital view.

**Figure 3:**
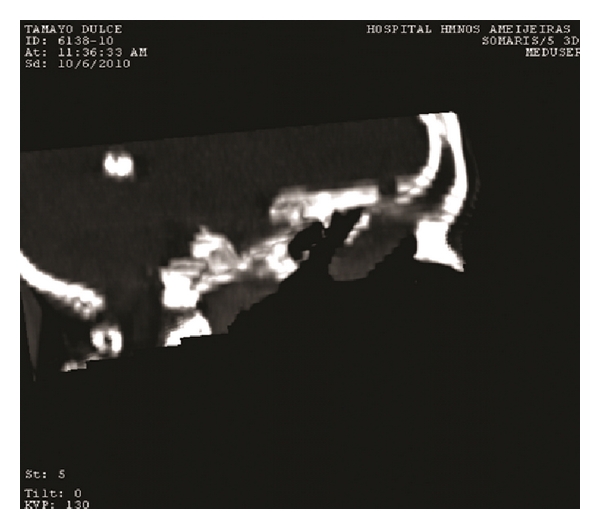
CT scan.

Our case is a 55-year-old female patient, who 23 years ago underwent transsphenoidal surgery for an intrasellar cyst. Five years after the surgery, hydrocephalus with cerebral spinal fluid leakage occurred and it was resolved by the placement of a ventriculoperitoneal shunt. Computed tomography did not define the bone defect at the site of previous surgery at that time. The patient presented before us, two years ago, with recurrent profuse rhinorrhea and nasal obstruction. She was admitted in Neurosurgical Department with headache, fever and vomiting, and bacterial meningitis which was successfully treated with antibiotics was diagnosed. MRI revealed protrusion of the brain and meninges from the anterior cranial fossa to the upper right nasal cavity. The T1-weighted sequences showed downward herniation of the ethmoidal roof on the right nasal cavity and T2-weighted coronal, and sagital imaging confirmed a liquid-filled mass in the right ethmoidal sinus and nasal cavity projecting through a defect in the ethmoidal roof (Figures 1 and 2). Computed tomography confirmed these findings (Figure 3). 

**Figure 4:**
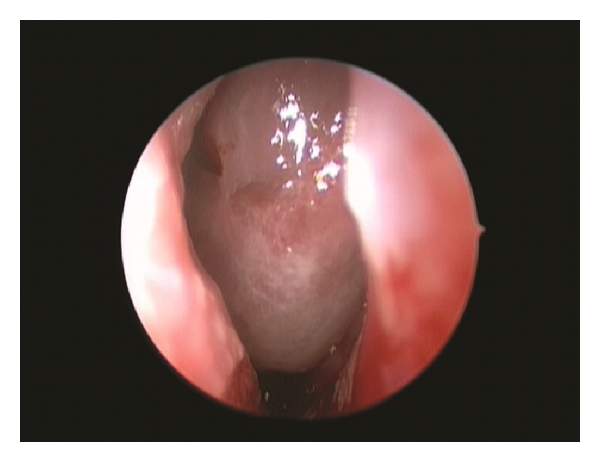
Endoscopic view of big meningoencephalocele within right nasal cavity.

**Figure 5:**
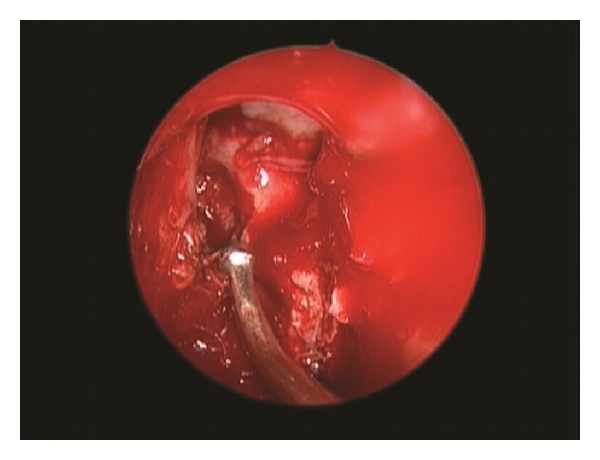
Endoscopic view of bony defect at the junction of ethmoidal sinus and posterior wall of the frontal bone.

An extended endonasal endoscopic approach to the anterior cranial base was practiced. A big meningoencephalocele within right nasal cavity was found (Figure 4). By using bipolar forceps the lesions were reduced. Middle turbinate was atrophic and middleward displaced. When the lesion was completely removed, a bony defect at the junction of ethmoidal sinus and posterior wall of the frontal bone were observed (Figure 5). This was reconstructed inlaying a free intradural fat graft and an epidural layer of bone, which was then covered with a nasoseptal flap. This was supported with balloon of 12 French Foley catheter in order to press the multilayer reconstruction against the defect. The patient had an uneventual recovery. There were no cerebrospinal rhinorrhea or any other complications and the patient was discharged six days after the surgery.

## 3. Discussion

A multidisciplinary management is recommended in the diagnosis and treatment of meningoencephalocele. Surgical procedures include removal and anterior fossa defect reconstruction by craniotomy and more recently endonasal endoscopic resection and repair or combination of these two techniques to remove the lesion and repair the defect [7–14]. 

Endonasal endoscopic procedures and special development of extended endonasal endoscopic approaches have increased the interest on treating this kind of lesion. That is why the treatment of meningoencephaloceles has become more popular. 

As the first step in the surgery, meningoencephalocele is carefully and gradually removed using bipolar cauterizing up to the level of the skull base. Then, mucosa surrounding skull-base defect is removed and the defect is prepared for the graft.

Selection of the graft material depends on the defected size and configuration, underlying pathophysiology and the surgeon's preference [15]. A multilayer repair is preferred not only to stop the CSF leak, but to reinforce the thin skull base and prevent meningoencephalocele recurrence [16, 17]. 

Autologous-free or pedicellate mucoperichondrial grafts, fascia, turbinate grafts, cartilage grafts, pericranial-galeal, and bone grafts have all been used successfully. Heterologus materials have also been used subdural as collagen matrix (Duragen, Integra Life Sciences) and no cells dermis grafts (Alloderm, Lifecell Corp.) [15]. Recently, reconstruction of skull base using vascularized pedicellar flap has been described. Nasoseptal and inferior turbinate flap seem to be the most useful. Reconstruction of the cranial base using vascularized tissue promotes fast and complete healing, preventing complications caused by persistent communication between the cranial cavity and the sinonasal tract [18–21]. 

We considered that best option for repairing the skull-base defect in this case was to use a multilayer grafts. We used a free inly fat graft (intradural), then an inlaid epidural bone graft, and, after that, we covered it with a big vascular pedicellar nasoseptal flap to prevent a recurrent brain herniation and CSF leakage. 

Historically, lumbar drainage of CSF has been used after surgery as an adjoined action to surgical repair. Nevertheless, the indications for a postoperative lumbar drainage have been debated and are very controversial in the literature [22, 23]. The lumbar drainage is used depending on the surgeon's preference. We have adopted the use of the blown balloon to support the multilayer reconstruction because it counteracts gravity and the pulsations of the brain, mitigates graft migration, and prevents development of a channel of fluid. It is usually removed at the fifth day. 

Surgical follow-up and postoperative care are always very important; it includes bed rest with the head elevated 30° for 2 weeks and the use of laxatives or stool softeners. In the absence of obvious infection the prevention with antibiotic is fully justified but further studies are necessary to prove its efficacy [24]. 

## 4. Conclusions

Endonasal endoscopic approach makes it possible for a direct work and total removal of ethmoidal meningoencephaloceles. A nasoseptal flap allows repairing the defect and practicing a watertight closure of the skull base reducing the rate of postoperative CSF fistulae.
